# A Descriptive Study of Brown Bear (*Ursus arctos*) Sperm Quality and Proteomic Profiles Considering Sperm Origin

**DOI:** 10.3390/ani15142064

**Published:** 2025-07-12

**Authors:** Marta Neila-Montero, Luis Anel-Lopez, Carolina Maside, Cristina Soriano-Úbeda, Rafael Montes-Garrido, Cristina Palacin-Martinez, Victoria Diez-Zavala, Santiago Borragán, Antonio Silva-Rodríguez, Francisco E. Martín-Cano, Luis Anel, Mercedes Alvarez

**Affiliations:** 1 Itra-ULE, INDEGSAL, University of León, 24071 León, Spain; mneim@unileon.es (M.N.-M.); cmasm@unileon.es (C.M.); msoru@unileon.es (C.S.-Ú.); rmong@unileon.es (R.M.-G.); cpalm@unileon.es (C.P.-M.); vdiez@unileon.es (V.D.-Z.); laner@unileon.es (L.A.); mmalvg@unileon.es (M.A.); 2 Animal Reproduction and Obstetrics, Department of Veterinary Medicine, Surgery and Anatomy, University of León, 24071 León, Spain; 3 Anatomy, Department of Veterinary Medicine, Surgery and Anatomy, University of León, 24071 León, Spain; 4 Cabárceno Park, 39693 Obregón, Spain; sborragan@cantur.com; 5 Facility of Innovation and Analysis in Animal Source Foodstuffs, University of Extremadura, 10003 Cáceres, Spain; asilvaro@unex.es; 6 Laboratory of Equine Spermatology and Reproduction, Veterinary Teaching Hospital, University of Extremadura, 10003 Cáceres, Spain; femartincano@unex.es

**Keywords:** electroejaculation, epididymis, urethral catheterization, semen, spermatozoa, sperm proteome

## Abstract

The conservation of small and genetically vulnerable brown bear populations, such as the Cantabrian subpopulation in Spain, depends on developing species-specific assisted reproductive technologies and genetic resource banks. However, the lack of standardized sperm collection and cryopreservation protocols hinders their application. This study provides the first comparative analysis of sperm quality and proteomic profiles from three different origins: epididymal, pre-ejaculated, and ejaculated. Sperm quality parameters —motility and kinetic, viability, apoptosis, and oxidative stress— and protein expression were assessed. Although yields were similar, marked differences were observed in sperm quality and protein profiles. Sixty-three proteins involved in metabolism, stress response, and oxidative balance were differentially expressed depending on sperm origin. Epididymal sperm showed the highest viability and motility, lowest apoptosis, and a proteomic profile indicative of active spermatogenesis and enhanced oxidative stress defense. In contrast, ejaculated sperm had increased oxidative stress and reduced expression of metabolic proteins, while pre-ejaculated sperm exhibited lower motility, likely due to urine contamination and mitochondrial protein alterations, despite comparable viability and apoptosis. These findings offer novel insights into brown bear sperm biology and highlight the importance of sperm origin in developing optimized assisted reproduction strategies, ultimately supporting ex situ conservation efforts for this species.

## Simple Summary

The brown bear population in the Cantabrian mountains of Spain is small and genetically vulnerable, which makes its conservation a priority. One way to help preserve this species is by developing reproductive techniques that enable scientists to collect, store, and utilize bear sperm to support breeding programs. However, there is still limited information about which methods are most effective for collecting and preserving brown bear sperm. In this study, we compared the sperm obtained using three different methods: directly from the reproductive organs, collected just before ejaculation via urethral catheterization, and collected via ejaculation using electrical stimulation. We examined the activity of the sperm and analyzed the types of proteins present, which provides clues about the sperm’s condition. We found that the sperm collected from the reproductive organs were in the best condition, with higher survival rates, increased movement, and greater stress resistance. This information will help scientists determine the most effective methods for collecting and storing brown bear sperm, thereby enhancing global efforts in wildlife conservation.

## 1. Introduction

The brown bear (*Ursus arctos*) is classified worldwide as a least-concern species by the International Union for Conservation of Nature (IUCN) Red List of Threatened Species, as its global population remains stable and is even increasing in some areas. The largest populations are in Russia, the United States (mainly Alaska), and Canada [1]. However, in Central and Southern Europe, brown bear populations are small, fragmented, and highly vulnerable [2]. Consequently, the European Union lists brown bears as endangered (Council Directive 92/43/EEC, annexes II and IV), and Spanish law considers them at risk of extinction (Real Decreto 139/2011). Specifically, the Cantabrian brown bear is the only autochthonous bear population in the Iberian Peninsula, and might represent the last genetically pure lineage of the brown bear in the world [3]. According to the Fundación Oso Asturias, this population is currently estimated to be around 350 individuals, divided into two nuclei (eastern and western) in the northern Spanish mountains [4]. Although conservation measures have contributed to a recent population increase, the situation of the species remains critical due to the small size of both subpopulations and the high risk of inbreeding and genetic variability loss [5].

Genetic resource banks play a crucial role in preserving genetic diversity by enabling the long-term storage of gametes and embryos for potential use in recovery programs using assisted reproductive techniques [3]. Nevertheless, the development of these banks is hindered by the limited knowledge of optimal protocols for sperm and oocyte collection, assessment, preservation, and application [6]. Traditionally, these procedures have been performed based on previous experience in domestic species, but the wide variety of methods and the different results obtained when applied to other species highlight the need for species-specific protocols [7].

Various methods have been documented for sperm collection in wild animals, grouped into three main types: (1) repeatable techniques in trained or conditioned animals, which include the use of artificial vaginas and phantoms [8,9,10,11,12], induced spontaneous ejaculation [13], and the manual stimulation of the penis or accessory sex glands [14,15,16,17]; (2) repeatable methods in anesthetized or sedated animals, involving electroejaculation [18,19,20] or chemically induced ejaculation [21,22]; and (3) non-repeatable methods, such as sperm collection from the epididymis or vas deferens after castration, sterilization, or death [23,24,25]. In bears, electroejaculation under anesthesia has been the sperm extraction method of choice over the years in several species, like the giant panda (*Ailuropoda melanoleuca*) [26,27], the Asiatic black bear (*Ursus thibetanus*) [28,29], the American black bear (*Ursus americanus*) [30], the Malayan sun bear (*Helarctos malayanus*) [31], the brown bear (*Ursus arctos*) [6,32,33], and the polar bear (*Ursus maritimus*) [34]. However, this technique presents some issues that should be considered, including expensive equipment, sudden arousal during anesthesia (with safety hazards), and the potential for urine contamination in the collected samples [35,36,37,38]. Moreover, the electroejaculation response can vary between species or males from the same species, and even between collections from the same male [39]. To overcome these shortcomings, urethral catheterization has been explored as an alternative method for sperm collection in polar bears (*Ursus maritimus*) [40] and Asiatic black bears (*Ursus thibetanus*) [38]. This technique utilizes medetomidine, a potent α2-adrenergic agonist that induces the smooth muscle contraction of the vas deferens, facilitating semen release into the urethra [41], but the quality of the sperm obtained via this method in bears remains poorly characterized. Additionally, as wild animals, valuable bears can die unexpectedly, making the collection of epididymal sperm the only way to preserve their gametes [42]. Despite its importance, little information exists on bear epididymal sperm cryopreservation [43].

Thus, this study aimed to describe and compare the quality and proteomic profiles of brown bear (*Ursus arctos*) sperm from three different origins (epididymal, pre-ejaculated, and ejaculated) as a first step in developing adapted cryopreservation protocols that would help to create biological resource banks for this species.

## 2. Materials and Methods

### 2.1. Reagents and Media

All chemicals used in this study were acquired from Sigma-Aldrich (Saint Louis, MI, USA), except the fluorescence probes Zombie Violet™ Fixable Viability Kit and CellEvent™ Caspase-3/7 Green Detection Reagent, which were purchased from BioLegend (San Diego, CA, USA) and ThermoFisher (Waltham, MA, USA), respectively.

### 2.2. Animals

A total of 14 sexually mature male brown bears were the subjects of the experiments during the breeding season (end of April to early July) from 2022 to 2024. Animals were housed in a half-freedom regime in Cabárceno Park (Cantabria, Spain; 43° 21′ N, 3° 50′ W; altitude 143 m) and fed a diet of chicken meat, bread, and fruits. Pre-ejaculated and ejaculated samples were obtained in one session, while epididymal sperm was collected from the same individuals in another session as part of a population control program in the park.

### 2.3. Sperm Collection

Males were immobilized via teleanesthesia with 750 mg of zolazepam HCl + tiletamine HCl (Zoletil 100^®^; Virbac, Carros, France) and 6 mg of medetomidine (Zalopin^®^; Orion Pharma Animal Health, Espoo, Finland). Animals were weighed and monitored during anesthesia (pulse, peripheral oxygen saturation, and breathing).

#### 2.3.1. Epidydimal Sperm

The testicular region of 12 brown bears was shaved, washed, and disinfected, and the males were castrated or epididectomized. Epididymides were dissected and cleaned of connective tissue and superficial blood vessels to avoid contamination. Sperm were collected through several incisions on the cauda epididymis with a surgical blade, and the emerging fluid was deposited in 0.5 mL graduated microtubes (*n* = 12). Sperm yield and functionality evaluations were performed at this moment.

#### 2.3.2. Pre-Ejaculated and Ejaculated Sperm

The pubic region of 14 brown bears was cleaned, the penis was washed with sterile physiological saline, and the rectum was emptied of feces. The bladder was catheterized to prevent the urine contamination of ejaculated samples, and it was possible to obtain a pre-ejaculate sperm sample from the urinary catheter in some bears (*n* = 12). After this, electroejaculation was performed with a PT Electronics electroejaculator (PT Electronics, Boring, OR, USA) and a 320 mm-long and 26 mm-wide transrectal probe. Electric stimuli (10 V and 250 mA on average) were given until ejaculation (*n* = 14). Ejaculated sperm samples were collected in 15 mL graduated glass tubes as isolated fractions. Osmolarity was measured using a cryoscopic osmometer OSMOMAT 3000 (Gonotec, Berlin, Germany), and subjective motility was assessed with a phase-contrast microscope (100× magnification) (Eclipse E400; Nikon, Tokyo, Japan). Fractions with a similar quality of the same electroejaculation were mixed and constituted one ejaculate. Pre-ejaculated and ejaculated sperm samples with a reduced concentration (<150 × 10^6^ sperm/mL) were centrifuged at 600× *g* for 6 min at room temperature, and the pellets were processed for sperm yield and functionality evaluations.

### 2.4. Sperm Yield Evaluation

Immediately after sperm collection from the three origins, the volume was recorded via the graduation marks of the collection tubes, the sperm concentration was analyzed using a cell counter (NucleoCounter SP-100; ChemoMetec, Allerod, Denmark), and the total sperm output was calculated by multiplying the measured volume by the corresponding sperm concentration.

### 2.5. Sperm Functionality Evaluation

#### 2.5.1. Sperm Motility and Kinetic Parameters

Sperm motility and kinetic parameters were assessed through the Computer-Assisted Sperm Analysis (CASA) Sperm Class Analyzer^®^ (SCA^®^), software version 6.4.0.89 (Microptic S.L., Barcelona, Spain). The system was set to capture a total of 50 frames at 100 frames/s and particles with an area of 12–62 µm^2^. Samples were diluted to 25 × 10^6^ sperm/mL in a TES-Tris-Fructose extender with 1% clarified egg yolk (320 mOsm/kg, pH 7.2) and pre-warmed for 5 min on a 37 °C plate. A 5 µL drop from each diluted sample was then placed into a Makler chamber (10 µm depth; Sefi Medical Instruments, Haifa, Israel) and analyzed on a warmed plate (37 °C) at a 100× magnification with a phase-contrast microscope and a BASLER acA1300-200uc digital camera (Basler Vision Technologies, Ahrensburg, Germany). At least 400 sperm from four randomly selected fields were captured and analyzed, removing non-sperm events. The kinetic parameters reported included linearity (LIN, %) and the amplitude of lateral head displacement (ALH, μm). Sperm exhibiting curvilinear velocities (VCLs) above 15 μm/s and 45 μm/s were classified as motile (total motility, TM) and progressively motile (progressive motility, PM), respectively.

#### 2.5.2. Sperm Viability and Apoptosis

Aliquots containing 2 × 10^6^ sperm were washed with phosphate-buffered saline (PBS) (300 mOsm/kg, pH 7.2) via brief centrifugation (15 s; MiniSpin plus, Eppendorf, Hamburg, Germany), and the supernatant was discarded. The resulting pellets were then stained at room temperature for 30 min in the dark with Zombie Violet™ Fixable Viability Kit (plasma membrane integrity probe) (1:1000 final dilution in PBS) and CellEvent™ Caspase-3/7 Green Detection Reagent (apoptosis marker) (4 µM final concentration in PBS). After that, another washing step was performed to stop cell staining, and the stain was fixed in 500 µL of 4% formaldehyde (ThermoFisher, Waltham, MA, USA) at room temperature and in darkness for 15 min. A new washing step was carried out, and the sperm pellets were resuspended in 1 mL of PBS. The samples were stored at 5 °C until delayed flow cytometry analysis, which occurred approximately four hours after staining and fixation upon arrival at the laboratory.

Flow cytometry analyses were conducted in a CytoFLEX S (Beckman Coulter, Brea, CA, USA) equipped with four lasers emitting at 405 (violet), 488 (blue), 561 (yellow), and 638 nm (red). A total of 40,000 events per sample and at least 20,000 sperm were recorded at 200–300 cells/s flow rate. Violet and green fluorescence were detected in FL5 (excitation 405 nm, emission 450/45 band pass (BP) filter) and FL1 (excitation 488 nm, emission 525/40 BP), respectively. Data were processed using FlowJo™ software version 10.8.1 (Ashland, Wilmington, DE, USA). Sperm viability was determined by the proportion of cells exhibiting low fluorescence with Zombie Violet™, indicating intact plasma membranes. Apoptotic cells were identified by their positive staining with CellEvent™ Caspase-3/7 Green, which marks caspase 3 and 7 activity. The gating strategy used has been previously described in detail by Palacin-Martinez et al. [44].

#### 2.5.3. Sperm Oxidation–Reduction Potential

The oxidation–reduction potential of the sperm samples was measured using the RedoxSYS™ diagnostic system (Luoxis Diagnostics, Englewood, CO, USA), a potentiometric device designed to evaluate the redox status of biological fluids or cell suspensions. To this end, 1 × 10^6^ sperm from each sperm sample were washed with 100 µL of PBS to remove debris, and the resulting sperm pellet was resuspended in 20 µL of PBS. After that, this volume (20 µL) was applied to the system’s disposable sensor, which measures electron transfer in response to the redox environment of the sample. The RedoxSYS™ system provided two key parameters within 4 min: the static ORP index (sORP), the integrated balance of oxidants and reductants reported in millivolts (mV); and the capacitance ORP index (cORP), the amount of antioxidant reserves expressed in microcoulombs (µC).

#### 2.5.4. Sperm Proteome

Sperm samples were centrifuged at 10,000× *g* for 15 min at 4 °C. The supernatant was discarded, and the resulting pellets (200 × 10^6^ sperm) were stored at −80 °C until further analysis. Phase-contrast microscopy was used to verify sample purity. The protein extraction, quantification, and digestion procedures followed the methodology previously established by Martín-Cano et al. [45]. Briefly, sperm were lysed in 400 µL of Protein Extraction Reagent Type 4 (7.0 M urea, 2.0 M thiourea, 40 mM Trizma^®^ base, and 1.0% C7BzO; pH 10.4) and incubated for one hour at 4 °C under constant agitation. Samples were then centrifuged at 17,000× *g* for 30 min at room temperature to remove cell debris, and the supernatant was collected. The protein concentration was determined using the 2-D Quant Kit (GE Healthcare, Sevilla, Spain), and 100 µg of protein from each sample was subjected to in-solution digestion with trypsin. Samples were analyzed in duplicate with an Agilent 1290 Infinity II UHPLC system coupled to an Agilent 6550 Q-TOF Mass Spectrometer via an Agilent Jet Stream Dual electrospray interface (Agilent Technologies, Santa Clara, CA, USA). Instrument control was managed using the MassHunter Workstation Data Acquisition software (Rev. B.06.01; Agilent Technologies, Santa Clara, CA, USA). Peptides (75 µg per sample) were injected into an Agilent AdvanceBio Peptide Mapping UHPLC column (2.7 µm, 150 × 2.1 mm; Agilent Technologies, Santa Clara, CA, USA) maintained at 55 °C with a constant flow rate of 0.4 mL/min. Chromatographic separation was achieved using a multi-step gradient: first, 2% of buffer B (water/acetonitrile/formic acid, 10:89.9:0.1) for 5 min, increasing to 45% over 40 min, followed by a ramp up to 95% B in 15 min, held constant for an additional 5 min. This 65 min run was followed by 5 min of using the initial condition for re-conditioning the column for the next run. The mass spectrometer operated in positive mode, with a nebulizer gas pressure of 35 psi, a drying gas flow of 10 L/min at 250 °C, and a sheath gas flow of 12 L/min at 300 °C. The capillary spray voltage was set at 3500 V, the fragmentor at 340 V, and the octopole RF Vpp at 750 V. The profile data were acquired for both MS and MS/MS scans in the extended dynamic range mode with a mass range of 50–1700 *m*/*z*, and scan rates of 8 spectra/s for MS and 3 spectra/s for MS/MS. The Auto MS/MS mode was used, with precursor selection by abundance and a maximum of 20 precursors selected per cycle. A ramped collision energy was used with a slope of 3.6, and an offset of –4.8 was applied. The same ion was rejected after two consecutive scans.

Data processing and analysis were performed using Spectrum Mill MS Proteomics Workbench (Rev B.04.01; Agilent Technologies, Santa Clara, CA, USA). Briefly, the raw data were extracted under default conditions, with non-fixed or variable modifications, [MH] + 50–10,000 *m*/*z*, maximum precursor charge +5, retention time and *m*/*z* tolerance ±60 s, minimum signal-to-noise ratio (S/N) 25, and the identification of ^12^C signals. The MS/MS search was conducted against the appropriate and updated protein database (in this case, UniProt/Bear) with the following criteria: non-fixed modifications, carbamidomethylated cysteines, and tryptic digestion with five maximum missed cleavages were selected as variable modifications. The ESI-Q-TOF instrument was set to a minimum matched peak intensity of 50%, maximum ambiguous precursor charge of +5, monoisotopic masses, peptide precursor mass tolerance of 20 ppm, product ion mass tolerance of 50 ppm, and to calculate the reversed database scores. The auto validation employed an auto-threshold method, in which the peptide score is automatically optimized for a target False Discovery Rate (FDR) of 1.2%. Subsequently, protein polishing validation was performed to increase the sequence coverage of the validated results, with a new maximum target FDR set at 0%.

### 2.6. Bioinformatic Analysis of Proteomic Data

The software R version 4.1.2 (Aukland, New Zealand) was used to perform all bioinformatic analyses. The data were quantile normalized. Differentially enriched proteins among sperm origins (epididymal, pre-ejaculated, and ejaculated) were identified using linear models (limma version 3.50.3), and *p* values were adjusted for multiple comparisons by applying Benjamini–Hochberg correction. Heatmaps were generated using the heatmap3 version 1.1.9 package. Proteins identified in the different sperm origin samples were queried against the Ursidae database in String (https://string-db.org/, accessed on 3 February 2025) to identify the biological pathways likely to be active in brown bear sperm.

### 2.7. Statistical Analysis

Statistical analyses of sperm yield and quality were performed and graphs were made using Prism 10 (GraphPad Software, San Diego, CA, USA). The homogeneity and normality of variables were examined via Levene’s and Kolmogorov–Smirnov tests. Normally distributed data were analyzed using one-way ANOVA, whereas non-normally distributed data were analyzed using the Kruskal–Wallis test. The results are displayed as the mean ± SEM (Standard Error of the Mean). Differences were statistically significant at *p* < 0.05.

## 3. Results

### 3.1. Sperm Collection Yield

The sperm collection yield data are shown in **Table 1**. The successful sperm collection rate (number of obtained sperm samples/number of attempts at sperm collection) was similar (*p* ≥ 0.05) for all sperm origins. However, the sperm sample volume obtained was significantly different (*p* < 0.05) among all sperm origins, with the lowest value for epididymal samples and the highest for ejaculated samples. The sperm concentration showed an opposite pattern to that of the volume, being significantly higher (*p* < 0.05) in epididymal samples and significantly lower (*p* < 0.05) in ejaculated samples compared to pre-ejaculated samples, which showed an intermediate result. Finally, the total sperm production was non-significantly different (*p* ≥ 0.05) among sperm origins.

### 3.2. Sperm Functionality Evaluation

#### 3.2.1. Sperm Motility and Kinetic Parameters

**Figure 1:**
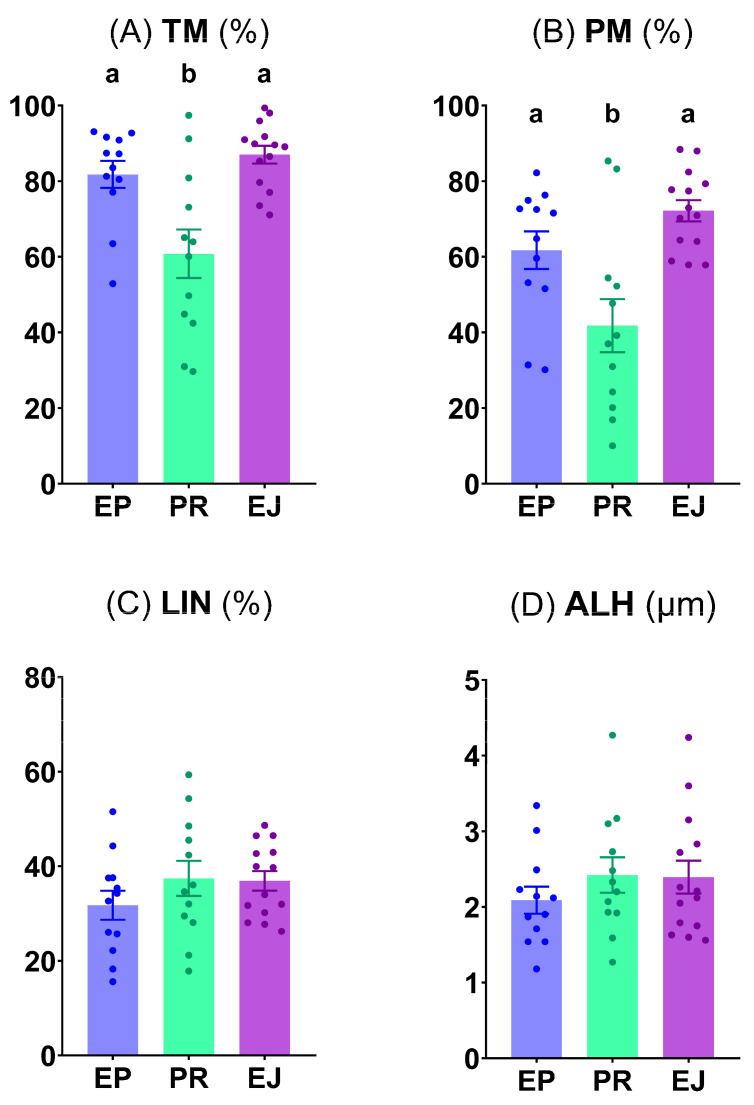
Motility and kinetic evaluation of brown bear sperm. (**A**) Total motility (TM, %); (**B**) Progressive motility (PM, %); (**C**) Linearity (LIN, %); and (**D**) Amplitude of lateral head displacement (ALH, µm). Graph dots represent the individual values of each male. Different lowercase letters (a, b) indicate significant differences (*p* < 0.05) among the different sperm origins (EP, epididymal; PR, pre-ejaculated; and EJ, ejaculated).

Pre-ejaculated sperm samples showed significantly lower (*p* < 0.05) TM and PM than the other two sperm origins (epididymal and ejaculated) **(Figure 1A,B)**. Nevertheless, the LIN and ALH were similar among the different sperm origins **(Figure 1C,D)**.

#### 3.2.2. Sperm Viability and Apoptosis

**Figure 2:**
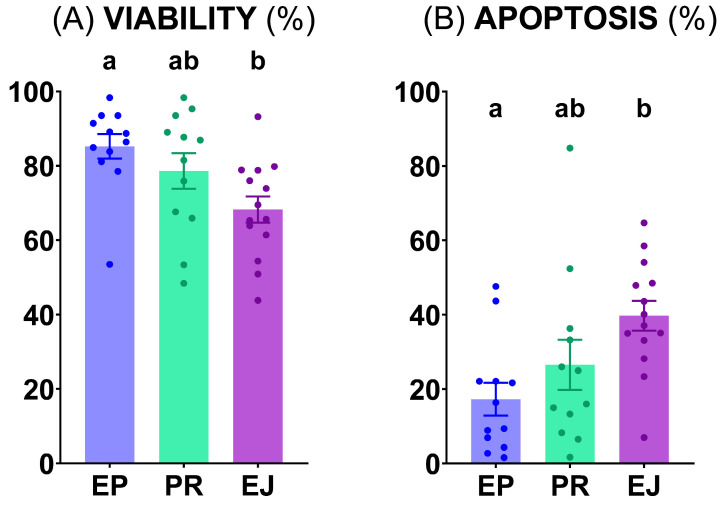
Flow cytometry evaluation of brown bear sperm. (**A**) Viable sperm (%) (Zombie Violet™); (**B**) Apoptotic sperm (%) (CellEvent™ Caspase-3/7 Green). Graph dots represent the individual values of each male. Different lowercase letters (a, b) indicate significant differences (*p* < 0.05) among the different sperm origins (EP, epididymal; PR, pre-ejaculated; and EJ, ejaculated).

Both sperm quality parameters determined via flow cytometry were significantly different (*p* < 0.05) in the ejaculated sperm samples compared to the epididymal ones. Specifically, sperm viability was significantly lower (*p* < 0.05) and sperm apoptosis was significantly higher (*p* < 0.05) in ejaculated sperm than in those obtained from epididymis **(Figure 2)**.

#### 3.2.3. Sperm Oxidation–Reduction Potential

**Figure 3:**
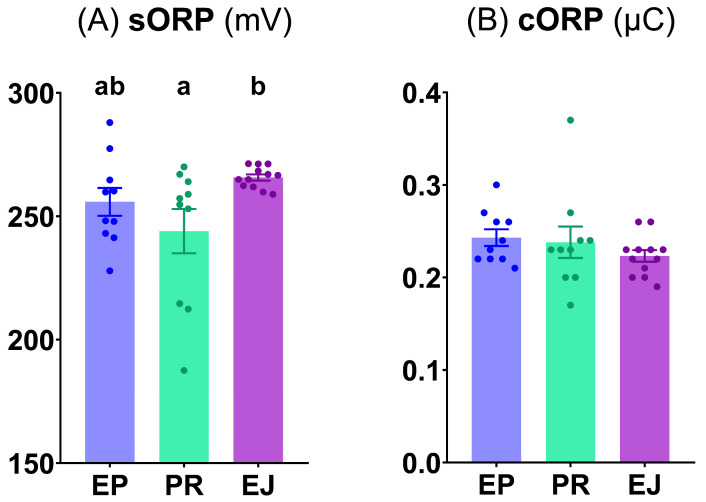
RedoxSYS evaluation of brown bear sperm. (**A**) Static ORP index (sORP, mV); (**B**) Capacitance ORP index (cORP, µC). Graph dots represent the individual values of each male. Different lowercase letters (a, b) indicate significant differences (*p* < 0.05) among the different sperm origins (EP, epididymal; PR, pre-ejaculated; and EJ, ejaculated).

Although the cORP index appeared similar (*p* ≥ 0.05) among the different sperm origins (epididymal, pre-ejaculated, and ejaculated) **(Figure 3B)**, the sORP index was significantly higher (*p* < 0.05) in the ejaculated samples than in the pre-ejaculated samples, with non-significant differences (*p* ≥ 0.05) between the epididymal sperm samples and the other two origins **(Figure 3A)**.

#### 3.2.4. Sperm Proteome

**Figure 4:**
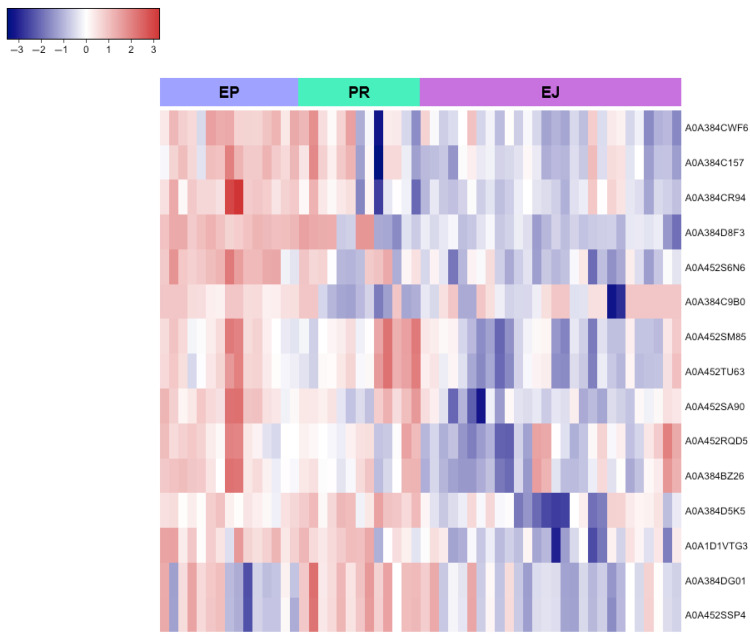
Heatmap showing the impact of sperm origin on the brown bear sperm. Proteins are classified following hierarchical clustering. Rows correspond to individual proteins (labeled with their UniProt IDs), and columns represent samples from each sperm origin (EP, epididymal; PR, pre-ejaculated; and EJ, ejaculated). The color scale indicates normalized expression levels, with red areas representing larger amounts of protein and blue regions representing smaller amounts of protein. Proteins were normalized and filtered by a fold change > 1 with *q* < 0.05.

**Figure 5:**
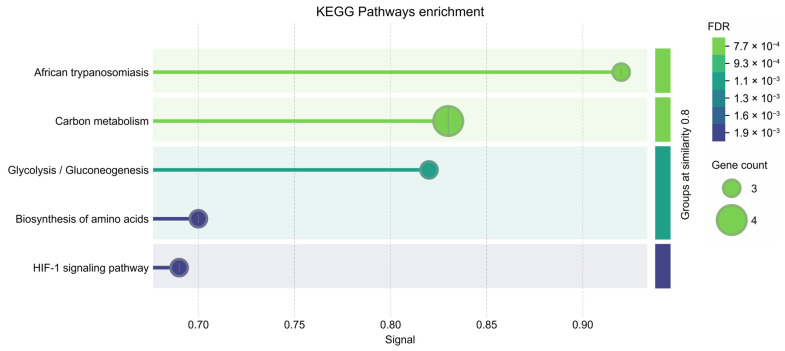
KEGG pathway enrichment analysis of under-expressed proteins in ejaculated brown bear sperm. Pathways are sorted by signal strength to highlight the most biologically relevant terms. Circle size represents the number of proteins associated with each path, while the color gradient indicates the false discovery rate (FDR), with green colors representing lower FDR values and blue colors having higher FDR values.

Changes in the relative amounts of proteins because of the sperm origin are presented as a heatmap **(Figure 4)**. A total of 63 proteins with significantly different expressions were identified among the different sperm origins (epididymal, pre-ejaculated, and ejaculated) with a *q*-value < 0.05 and a fold change > 1. Interestingly, proteins related to *African trypanosomiasis, carbon metabolism, glycolysis/gluconeogenesis, the biosynthesis of amino acids*, and *the HIF-1 signaling pathway* were decreased in the ejaculated sperm **(Figure 5)**.

**Figure 6:**
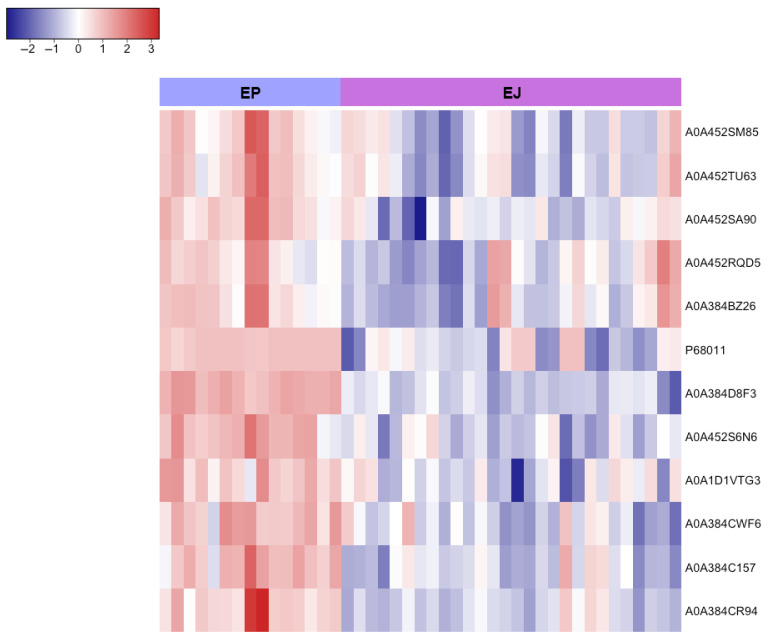
Heatmap representing the differential protein expression between epididymal (EP) and ejaculated (EJ) brown bear sperm. Proteins are classified following hierarchical clustering. Rows correspond to individual proteins (labeled with their UniProt IDs) and columns represent samples from each sperm origin. The color scale indicates normalized expression levels, with red areas representing larger amounts of protein and blue regions representing smaller amounts of protein. Proteins were normalized and filtered by a fold change > 1 with *q* < 0.05.

**Figure 7:**
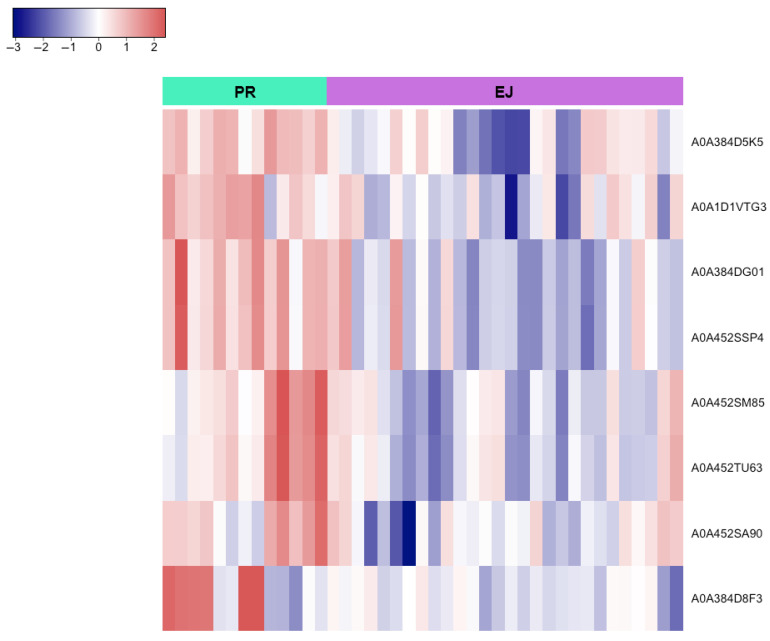
Heatmap representing the differential protein expression between pre-ejaculated (PR) and ejaculated (EJ) brown bear sperm. Proteins are classified following hierarchical clustering. Rows correspond to individual proteins (labeled with their UniProt IDs) and columns represent samples from each sperm origin. The color scale indicates normalized expression levels, with red areas representing larger amounts of protein and blue regions representing smaller amounts of protein. Proteins were normalized and filtered by a fold change > 1 with *q* < 0.05.

Moreover, a pairwise analysis of sperm origins (epididymal vs. ejaculated and pre-ejaculated vs. ejaculated) identified the proteins shown in **Figure 6** and **Figure 7** as having the lowest abundance in the ejaculated sperm samples, as well as the proteins whose expression is primarily associated with just one sperm origin **(Table 2)**.

## 4. Discussion

Sperm collection methods directly impact sperm quality and usability in assisted reproductive technologies, making them an essential consideration in wildlife conservation programs [7]. Although electroejaculation has historically been the preferred method in bears, its limitations make it necessary to explore alternative approaches [38,40]. To our knowledge, this is the first study evaluating the quality and proteomic profiles of brown bear (*Ursus arctos*) sperm of three different origins (epididymal, pre-ejaculated, and ejaculated), contributing to the development of species-specific sperm manipulation and cryopreservation protocols.

From a sperm yield perspective, our results indicated that the three techniques were similar for the brown bear, as no differences were observed in the collection success or the total sperm output. These findings contrast with those reported by other researchers and suggest that electroejaculation appears to be more effective in brown bears (90.5–100% success rate and 545.0 ± 51.9–1387.2 ± 2160.7 × 10^6^ sperm, based on the data of [46] and that of Ishikawa et al. [33]) than in other bear species, such as the Asiatic black bear (*Ursus thibetanus*) (53.8% success rate and 100.9 ± 70.0 × 10^6^ sperm) [38], the American black bear (*Ursus americanus*) (53.8% success rate, no sperm production data) [30], or the polar bear (*Ursus maritimus*) (16.7% success rate and estimated 10.1 × 10^6^ sperm) [34]. This difference is probably due to the presence of adipose tissue surrounding the internal organs in species adapted to colder environments, which is non-conductive and increases electrical impedance, reducing electroejaculation’s efficiency [47]. In contrast, the pre-ejaculated sperm collection method in our study yielded outcomes (85.7% success rate and 1089.0 ± 283.9 × 10^6^ sperm) comparable to those reported for other bear species where this technique has been employed (92.3% success rate and 1196.6 ± 955.5 × 10^6^ sperm in the Asiatic black bear, and 72.0% success rate with total sperm count data not provided in the polar bear) [34,38]. Sperm collection via urethral catheterization is highly reliant on the adrenergic effects of the anesthetic drugs used, which are effective in a wide range of domestic [22,48,49,50] and wild felines [21,51,52], dogs [53], foxes [54], ferrets [55,56], macaques [57], rhinoceros [58], and so on. Finally, in epididymal sperm collection, we also achieved better results than those reported by previous authors, with sperm successfully recovered from 100% of the males and a sperm production of 509.6 ± 116.2 × 10^6^ sperm. In contrast, Wojtusik et al. [34] reported successful sperm rescue in only 57.1% of collections in polar bears without sperm concentration data. These differences may be attributed to our use of apparently healthy individuals as part of a reproductive control program, whereas the other researchers opportunistically sampled animals that had died naturally or were euthanized due to illness. Similarly, Archibald et al. [59] successfully collected epididymal sperm via mincing in 78.6% of American black bears without reporting sperm production data, but these animals were legally killed by hunters outside the breeding season, so the lower success rate could be related to the timing of sperm collection.

**Figure A1:**
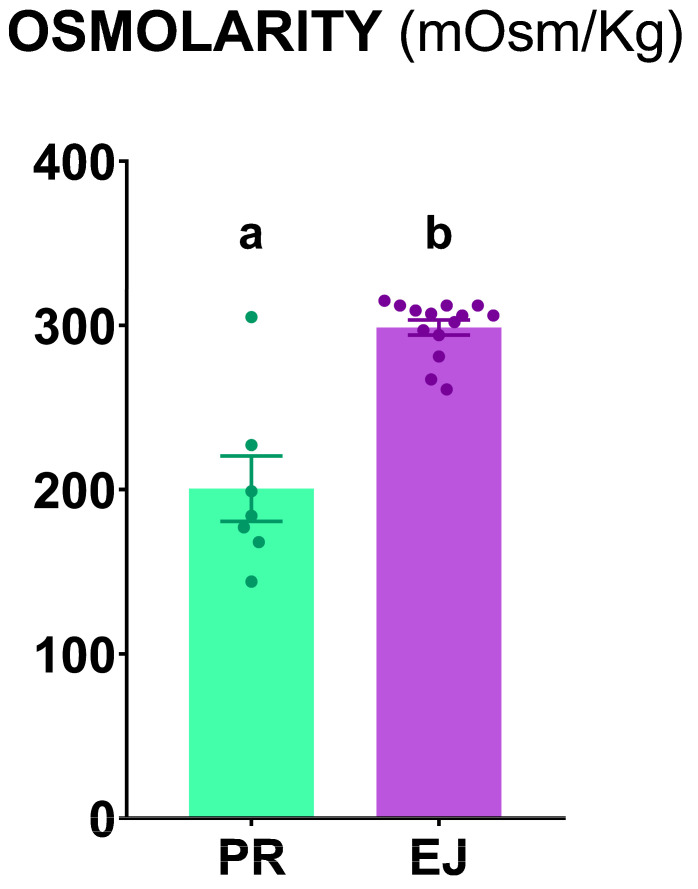
Osmolarity of brown bear sperm samples (mOsm/Kg). Graph dots represent the individual values of each male. Different lowercase letters (a, b) indicate significant differences (*p* < 0.05) among the different sperm origins (PR, pre-ejaculated; EJ, ejaculated).

Concerning sperm quality, both the total and progressive motility of brown bear sperm displayed their lowest values in the pre-ejaculated samples, in contrast to other authors’ observations in similar studies on different species. In Asiatic black bears [38] and rhinoceros [58], no differences were found in the sperm motility status between samples obtained via urethral catheterization and those collected via electroejaculation. On the other hand, Prochowska et al. [49] noticed a higher total motility in fresh urethral sperm from domestic cats compared to fresh epididymal sperm, with no differences in other motility or kinetic parameters assessed in their study. Despite the above, the motility values of brown bear epididymal and ejaculated sperm are consistent with the existing literature on this species [43,46,60,61,62], and we associate the reduced motility of the pre-ejaculated sperm samples with the presence of urine, as reflected by the lower osmolarity in these samples compared to the ejaculated ones **(Figure A1)**. Urine contamination in urethral catheterization sperm collections has been previously reported by Wojtusik et al. [34] in polar bears, linked to a rapid loss of motility and viability after collection. Similar findings have been described in wolves [63] and dogs [53], affecting up to 44% of samples and reducing motility and progressive movement. However, the pre-ejaculated samples in our study did not differ from the other two sperm origins in terms of viability and apoptosis, although the sperm quality was lower for both parameters in the ejaculated samples than in the epididymal samples, as has been documented in other species due to differences in the sperm proteins involved in metabolism, stress response, and redox balance [64,65]. In this context, the RedoxSYS system is a novel technology that measures the static (sORP) and capacity (cORP) oxidation–reduction potential, with sORP referring to the passive or current state of activity between oxidants and antioxidants [66,67]. Brown bear sperm samples of different origins showed similar cORP values, but ejaculates exhibited the highest sORP index, indicating greater oxidative stress in these samples.

Using proteomic strategies, 63 proteins with differential expression were identified among the different sperm origins (epididymal, pre-ejaculated, and ejaculated). Enrichment analysis revealed that pathways such as *African trypanosomiasis, carbon metabolism, glycolysis/gluconeogenesis, the biosynthesis of amino acids,* and *HIF-1 signaling* were under-expressed in ejaculated brown bear sperm compared to epididymal and pre-ejaculated sperm. Sperm are highly specialized cells whose primary function is to deliver the male genetic material to the oocyte. Given their specialization and function, sperm exhibit high metabolic activity and require more energy than other cells [68], so they can use both exogenous and endogenous carbohydrates, as well as other carbon-containing compounds such as lactate and pyruvate, to meet their energy demand via two main pathways: glycolysis (anaerobic pathway) and oxidative phosphorylation (aerobic pathway) [69,70]. Although the machinery for both processes is present in sperm from all species, the predominant ATP generation route appears to be species-specific [71]. Little is known about sperm metabolism in wildlife and endangered species, where the impact of inbreeding on sperm function or the need to preserve genetic material requires knowledge of their metabolism, since it is relevant to sperm performance and use in assisted reproductive techniques [72]. In this context, the identification of key glycolytic enzymes such as *Glyceraldehyde-3-phosphate dehydrogenase* and *Pyruvate kinase* suggests that glycolysis may play a major role in energy production and the maintenance of essential metabolic functions in brown bear sperm. Notably, these proteins were less active in ejaculated sperm than in epididymal and pre-ejaculated sperm. This finding is surprising and contrasts with results from bovine sperm reported by Zoca et al. [65], where energy production, particularly through glycolysis, is more regulated in the epididymis than after ejaculation. Indeed, it is well established that sperm in the caudal epididymis remain in a quiescent state due to the presence of both quiescence and anti-premature sperm activation factors in the epididymal fluid [73,74,75], which dilute at ejaculation by mixing with seminal plasma, leading to an increase in sperm metabolism and motility [76]. Regarding amino acid biosynthesis, our results are in agreement with those of Setchell et al. [77], who reported that the concentration of the amino acid glutamate in epididymal fluid was up to ten times higher than in testicular fluid and seminal plasma in rams. Lastly, the hypoxia-inducible factor-1 (HIF-1) signaling pathway is recognized as a key mediator of cellular adaptation to hypoxia [78,79]. Therefore, it appears normal that its activity was higher in epididymal and pre-ejaculated brown bear sperm. As sperm transit from the testis to the distal epididymal regions, they are thought to encounter a gradually increasing oxygen concentration within the luminal microenvironment [80]. In this situation, sperm produce reactive oxygen species (ROS) and are highly susceptible to ROS oxidative damage, which may activate HIF-1 [81]. Alternatively, certain areas of the epididymis may exhibit localized hypoxia. In particular, the distal epididymis exhibits a less dense capillary network compared to the proximal segments [82], which may limit oxygen delivery [83]. Moreover, the increased luminal diameter of the cauda epididymis contributes to greater diffusion distances for oxygen, potentially resulting in small local fluctuations in oxygen availability [84]. These conditions, combined with the large number of sperm stored in this region, may demand enhanced protective mechanisms to cope with variable oxygen tensions. It has also been suggested that localized hypoxia may exist within densely packed sperm populations in the epididymal tail [81], which could be another reason for the activation of HIF-1 in the brown bear epidydimal and pre-ejaculated sperm. This information could guide the design of cryopreservation media or pre-freezing treatments aimed at enhancing sperm resilience to freezing-induced damage, for example, through antioxidant supplementation or hypoxia-mimicking conditions. Further studies should explore whether the metabolic and molecular profiles observed in these sperm types confer better post-thaw quality, which would be particularly relevant for germplasm banking and assisted reproduction in endangered wildlife.

A more detailed pairwise proteomic comparison between sperm origins (epididymal vs. ejaculated) identified the following proteins as predominantly expressed in epididymal sperm from brown bears: *Heat shock-related 70 kDa protein 2*; *Heat shock cognate 71 kDa protein*; *78 kDa glucose-regulated protein*; *Phosphoglycerate kinase (PGK1)*; *Actin, cytoplasmic 2*; and *Actin, aortic smooth muscle*. Heat Shock Proteins (HSPs), particularly those belonging to the 70 kDa family (HSP70), are commonly present on the sperm’s surface across various species [85,86]. Specifically, *Heat shock-related 70 kDa protein 2 (HSP70-2)* has been strongly associated with mammalian spermatogenesis [87]. This complex process involves the mitotic proliferation of spermatogonia, the meiotic progression of spermatocytes, and the post-meiotic transformation of spermatids into mature sperm [88]. In human germ cells, HSP70-2 expression occurs notably at two specific phases: (1) during meiosis, where it is a component of the synaptonemal complex, and (2) in the final stages of spermiogenesis, where it is involved in spermatid elongation [89]. In mice, HSP70-2 is essential for the progression of meiosis [90,91,92]. Moreover, HSP70-2 is crucial for sperm binding to the zona pellucida in humans [89], boars [93], and bulls [94], highlighting its important role in male fertility. The 78 *kDa glucose-regulated protein (GRP78)* is another key member of the HSP70 family closely related to HSP70-2, which shares over 61% of the amino acid sequence similarity with it. In mouse testis sections, GRP78 shows strong immunostaining from pachytene spermatocytes to post-meiotic germ cells, but is absent in spermatogonia and other cell types [95]. Wang et al. [96] also demonstrated that GRP78 expression in human and rat testes increased with age, and that its localization in different epididymal regions indicated a dynamic secretory model, suggesting its involvement in mammalian spermatogenesis. Numerous studies have shown that GRP78 expression is linked to resistance to apoptotic cell death in somatic cells, particularly in progressively growing tumors [97,98,99]. Like tumorigenesis, spermatogenesis is a dynamic and complex process involving extensive cell proliferation and differentiation, where apoptosis control is critical [100]. It plays a crucial role in limiting the testicular germ cell population during male development and eliminating germ cells with altered DNA at various phases of spermatogenesis [101,102]. In our study, the predominant expression of these two heat shock proteins in epididymal sperm supports their role in brown bear spermatogenesis. In turn, *Heat shock cognate 71 kDa protein (HSC71)*, a product of the HSP70 family, appears to play a role in the terminal differentiation of mammalian male germ cells and/or in the function of mature sperm, particularly through their ATPase activity [103,104]. Related to this protein, *Phosphoglycerate kinase (PGK)* is an enzyme that catalyzes ATP production within the glycolytic pathway [105]. PGK exists in two isoforms, PGK1 and PGK2, which support sperm function at different germ cell stages. PGK1, the isoform identified in this study, is primarily expressed in spermatogonial germ cells, while PGK2 is found in round spermatids, although low levels of PGK1 may persist after meiosis at levels sufficient for spermatid metabolism [106,107,108]. It is unusual to find spermatids or immature sperm in the epididymal tail, so we hypothesized that the presence of PGK1 in brown bear sperm cells collected from the epididymis could be linked to increased sexual activity during the breeding season, similar to findings reported in rams [109]. Conversely, PGK2 detection, as expressed at late stages of spermatogenesis, has been used to predict the presence of more mature sperm [110]. Finally, regarding the detection of actin in brown bear epididymal sperm samples, there are two different causes. *Cytoplasmic Actin 2* is involved in germ cell movement, cargo transportation, and nuclear modification during spermatogenesis [111], while *Smooth Muscle Actin* probably derives from the smooth muscle cells of the epididymal blood vessels due to tissue slicing for sperm retrieval [112].

Similarly, the pairwise comparison between pre-ejaculated and ejaculated sperm samples revealed only two proteins predominantly present in pre-ejaculated brown bear sperm: *Glutathione S-transferase* and *Isoaspartyl peptidase/L-asparaginase*. It has been well established that ROS play a dual role in sperm, having beneficial or harmful effects depending on their concentration, localization, or duration of exposure [113]. In the testis, the continuous division of germ cells is accompanied by high mitochondrial activity, leading to elevated ROS production that can negatively impact spermatogenesis [114]. *Glutathione S-transferases (GSTs)* are a large group of enzymes commonly implicated in controlling physiological ROS levels and protecting sperm and germ cells against oxidative stress by scavenging electrophilic substances [115]. Different GST isoforms have been previously identified in sperm [116], seminal plasma [117], and male reproductive tissues [118]. Although these enzymes generally operate as antioxidants in most mammalian cell types, sperm GSTs play a triple role: (1) in cell detoxification (preventing lipid membrane peroxidation and subsequent oxidative stress) [119], (2) in cell signaling regulation (involved in spermatogenesis and sperm capacitation) [120,121], and (3) in fertilization (since specific GST members are involved in sperm–oocyte binding and the acceleration of sperm nuclear decondensation) [122,123,124]. Thus, our findings support the relevance of GSTs in oxidative stress regulation during brown bears’ pre-ejaculatory stages. Conversely, the function of the *Isoaspartyl peptidase/L-asparaginase (ASRGL1)* remains unclear, although it is known to localize in the middle part of the sperm tail, probably associated with mitochondria [125]. Higher expression levels have been related to asthenozoospermia in humans [125,126], which, together with the lower osmolarity, could explain the reduced motility observed in the pre-ejaculated brown bear sperm samples.

## 5. Conclusions

To sum up, although the three sperm origins (epididymal, pre-ejaculated, and ejaculated) showed similar sperm yield results, this study highlights key differences in sperm quality and protein expression in the brown bear. Epididymal sperm exhibited superior quality and a proteomic profile consistent with active spermatogenesis and oxidative stress protection mechanisms, whereas ejaculated sperm showed higher oxidative stress and reduced expression of essential metabolic proteins. Pre-ejaculated sperm, while comparable in yield, demonstrated compromised motility, likely associated with urine contamination and mitochondrial protein alterations. These findings offer novel insights into brown bear sperm biology and highlight the importance of sperm origin in developing optimized cryopreservation protocols and assisted reproduction strategies, ultimately supporting ex situ conservation efforts for this species.

## Figures and Tables

**Table 1:** Sperm yield in brown bears using epididymal (EP), pre-ejaculated (PR), or ejaculated (EJ) collection methods.

SPERM ORIGIN	EP	PR	EJ
**Successful collection rate**	100% (12/12)	85.71% (12/14)	100% (14/14)
**Volume (mL)**	0.14 ± 0.03 ^a^	1.93 ± 0.62 ^b^	3.49 ± 0.34 ^c^
**Concentration (×10^6^ sperm/mL)**	3509.00 ± 324.60 ^a^	1399.00 ± 336.60 ^b^	191.30 ± 36.46 ^c^
**Total output (×10^6^ sperm)**	509.60 ± 116.20	1089.00 ± 283.90	602.60 ± 107.00

Different lowercase letters (a, b, and c) indicate significant differences (*p* < 0.05) among the different sperm origins.

**Table 2:** Under-expressed proteins in ejaculated brown bear sperm compared to epididymal (EP) and/or pre-ejaculated (PR) sperm.

SPERM ORIGIN		UniProt ID	Protein	Organism
**EP**		A0A384CWF6	Heat shock-related 70 kDa protein 2	Polar bear (*Ursus maritimus*)
		A0A384C157	Heat shock cognate 71 kDa protein	Polar bear (*Ursus maritimus*)
		A0A384CR94	78 kDa glucose-regulated protein	Polar bear (*Ursus maritimus*)
		A0A452S6N6	Phosphoglycerate kinase (PGK1)	American black bear (*Ursus americanus*)
		A0A452RQD5	Actin, cytoplasmic 2	American black bear (*Ursus americanus*)
		A0A384BZ26	Actin, aortic smooth muscle	Polar bear (*Ursus maritimus*)
	**PR**	A0A384D8F3	Clusterin	Polar bear (*Ursus maritimus*)
		A0A452SM85	Pyruvate kinase	American black bear (*Ursus americanus*)
		A0A452TU63		Polar bear (*Ursus maritimus*)
		A0A452SA90	Glyceraldehyde-3-phosphate dehydrogenase	American black bear (*Ursus americanus*)
		A0A384D5K5	Glutathione S-transferase	Polar bear (*Ursus maritimus*)
		A0A452SSP4	Isoaspartyl peptidase/L-asparaginase	American black bear (*Ursus americanus*)
		A0A384DG01		Polar bear (*Ursus maritimus*)

## Data Availability

The data presented in this study are available on request from the corresponding author.
